# *BRCA1/BRCA2* mutations in Japanese women with ductal carcinoma in situ

**DOI:** 10.1002/mgg3.493

**Published:** 2019-01-16

**Authors:** Yan Liu, Yoshimi Ide, Mayuko Inuzuka, Sakiko Tazawa, Yoko Kanada, Yuki Matsunaga, Takashi Kuwayama, Terumasa Sawada, Sadako Akashi‐Tanaka, Seigo Nakamura

**Affiliations:** ^1^ The 3rd Department of Breast Cancer, Tianjin Medical University Cancer Institute and Hospital National Clinical Research Center for Cancer Tianjin China; ^2^ Key Laboratory of Breast Cancer Prevention and Therapy of Ministry of Education Tianjin China; ^3^ Key Laboratory of Cancer Prevention and Therapy Tianjin China; ^4^ Tianjin's Clinical Research Center for Cancer Tianjin China; ^5^ Division of Breast Surgical Oncology, Department of Surgery Showa University School of Medicine Tokyo Japan; ^6^ Department of Pathology Showa University Hospital Tokyo Japan

**Keywords:** *BRCA 1/2* mutations, breast cancer, ductal carcinoma in situ, family history

## Abstract

**Background:**

Ductal carcinoma in situ (DCIS) is considered a component of the clinical spectrum of breast cancer even in those with *BRCA1/2* mutation. The aim of this study was to report the feature of DCIS raised in Japanese women with *BRCA1/2* mutations.

**Methods:**

A total of 325 Japanese women with breast cancer (BC) (with or without invasive cancer) were referred for genetic counseling and underwent genetic testing for mutations in the *BRCA1* and *BRCA2* genes in Showa University Hospital between December 2011 and August 2016. And 49 of them who were pathologically diagnosed as DCIS were included in this study. Logistic regression models were fit to determine the associations between potential predictive factors and *BRCA* status. A Cox proportional hazards model is used to predictive value of parameters for Ipsilateral breast tumor recurrence (IBTR) and contralateral breast tumor recurrence (CBTR).

**Results:**

(a) Of 325 patients (with or without invasive cancer), 19.1% (62/325) tested positive for *BRCA1*/*BRCA2* mutations. And 18.4% (9/49) was positive for *BRCA1*/*BRCA2* mutations in DCIS, compared with 19.2% (53/276) in IDC (*p *= 1.000). Among *BRCA* mutations, 14.5% (9/62) had DCIS compared with nonmutations (15.2%, 40/263). Incidence of DCIS was 3.0% (1/33) of *BRCA1* mutations and 27.5% (8/29) of *BRCA2* mutation (*p *= 0.009). (b) Median age of diagnosis in *BRCA* mutation carriers was 39 years, compared with 46 years in noncarriers. Age, Family history (FH) of BC, FH of first or second BC and total number of relatives with BC diagnosis (DX) has significant difference between *BRCA* mutation carriers and noncarriers in univariate analysis. In a multivariate logistic model, total relatives with BC DX ≥ 2 (odds ratio [OR], 5.128; 95% confidence interval [CI], 1.266–20.763; *p *= 0.022), age at diagnosis ≤35 years (OR 0.149, 95% CI 0.023–0.954, *p *= 0.045) and ER+/HER2+ status (OR 5.034, 95% CI 1.092–23.210, *p *= 0.038) remained as independent significant predictors for *BRCA* mutation. Ki67 index (cut off by 14% or 30%) did not differ between *BRCA* mutation carriers and noncarriers (*p *= 0.459 and *p *= 0.651). (c) There was a significant difference in ER‐positive tumors among *BRCA2* carriers and noncarriers (*p *= 0.042). Subgroup analysis showed *BRCA2* carriers tend to be of higher grade (Grade 2 and 3), more frequently ER+/PR+ (*p *= 0.041) and lower proliferation (Ki67 index) than noncarriers, whereas differences in nuclear grade and ki67 index were not found significantly in our study. (d) *BRCA* mutation was not associated with an increased risk of IBTR and CBTR.

**Conclusion:**

DCIS is equally as prevalent in patients who were *BRCA* mutation carriers as in high familial‐risk women who were noncarriers, but occurs at earlier age. *BRCA2* carriers have higher incidence in DCIS than that of *BRCA1* carriers, and tend to be higher grade and more frequently ER positive and lower proliferation. Total relatives with BC DX ≥2, age at diagnosis ≤35 years and ER+/HER2+ might be independent predictors for *BRCA* mutation in Japanese women with DCIS and patients of these risk factors should be recommended to receive genetic counseling and *BRCA* testing.

## 1 INTRODUCTION

Since 1996, breast cancer has become the most frequent malignancy in women in Japan and estimates of new cases in 2016 were 90,000 according to National Cancer Center of Japan. Widespread adoption of screening increases breast cancer incidence in a given population and changes the characteristics of cancers detected, with increased incidence of lower‐risk cancers, premalignant lesions, and ductal carcinoma in situ (DCIS). Although DCIS is not immediately life‐threatening, it is associated with an increased risk of invasive breast cancer (IBC). Pathologists agree that DCIS is a preneoplastic lesion, and shares certain features with IBC such as genetic signature and epidemiologic risk factors (Fujii, Szumel, Marsh, Zhou, & Gabrielson, 1996; Hwang et al., 2004; O'Connell et al., 1998).

The identification of deleterious mutations in the breast cancer susceptibility genes *BRCA1* and *BRCA2* has important implications for mutation carriers in general, because they are the principal cause of Hereditary Breast and/or Ovarian Cancer Syndrome (Miki et al., 1994; Wooster et al., 1995). Of all women with breast cancer, 5% to 10% may have a germline mutation of the genes *BRCA1* and *BRCA2* (Blackwood & Weber, 1998). The estimated lifetime risk of developing breast cancer for women with *BRCA1* and *BRCA2* mutations is 40% to 85%. Mutation carriers with a history of breast cancer have an increased risk of contralateral disease that may be as high as 5% per year (Frank et al., 1998). Mutations in either the *BRCA1* or the *BRCA2* gene also confer an increased risk of ovarian cancer (Easton et al., 1999; Ford, Easton, Bishop, Narod, & Goldgar, 1994) or other primary cancers (Easton et al., 1999; Ford et al., 1994). When *BRCA1*/*BRCA2* mutation carriers were diagnosed before age 40 years, the risk of a contralateral breast cancer (CBC) reached nearly 50% in the ensuing 25 years (Garber & Golshan, 2009; Graeser et al., 2009).

The association of *BRCA* mutations with IBC is well established. DCIS is now considered a component of the *BRCA* mutation clinical spectrum (Bayraktar et al., 2012; Hall, Reid, & Wenstrup, 2010; Hwang et al., 2007; Smith et al., 2007). A few retrospective studies have examined the prevalence of *BRCA1*/*BRCA2* mutations in women diagnosed with DCIS and have reported mutation rates ranging between 3.3% (Claus, Petruzella, Matloff, & Carter, 2005) and 13% (Frank et al., 2002; Hall, Reid, & Wenstrup, 2010). Those studies support the concept that women with DCIS, like their counterparts with IBC, warrant genetic risk assessment and testing on the basis of high‐risk variables. Notably, the knowledge of a *BRCA1*/*BRCA2* mutation is likely to significantly change the assessment of a DCIS patient's risks for future cancers and the cancer prevention/risk reduction recommendations that would be considered. Subsequent studies have led to the suggestion that the preinvasive phase may be shortened or even absent in hereditary breast cancers, particularly those associated with *BRCA1* mutations (Jacquemler, Eisinger, Guinebretiere, Stoppa‐Lyonnet, & Sobol, 1996). But patients in studies above are almost from US and other Western countries, while only a few reports have been published concerning the prevalence of *BRCA1*/*BRCA2* mutations with DCIS among Japanese people (Ikeda et al., 2001; Kawahara et al., 2004; Noguchi et al., 1999; Sekine et al., 2001).

The purpose of our study is to further characterize the association between DCIS and *BRCA* mutations in Japanese women and to provide the genetic basis for directing the treatment and predicting prognosis for DCIS women with *BRCA1/BRCA2* mutation. We also hope to find special independent predictors for *BRCA* mutation in DCIS and could recommend genetic counseling and testing for Japanese patients with DCIS of high risk of *BRCA* mutation.

## 2 MATERIALS AND METHODS

### 2.1 Patients

A total of 325 Japanese women with BC (with or without invasive cancer) were referred for genetic counseling and underwent genetic testing for mutations in the *BRCA1* and *BRCA2* genes in Showa University Hospital from December 2011 to August 2016. Each participant was either self‐ or physician‐referred to genetic counseling. And 49 of them who were pathologically diagnosed as DCIS were included in this study. Any patients with IBC, identified either at time of biopsy or after tumor removal surgery, were excluded. All pathologic specimens were reviewed by a dedicated breast pathologist at our institution and all patients underwent routine stage workup.

Demographic and clinical information were collected from the retrospectively maintained breast cancer research database under institutional review board‐approved protocols and included age at the time of diagnosis, family history (FH) of breast cancer (BC) and/or ovarian cancer (OC)in at least 1 first‐degree and/or second‐degree relative, number of relatives affected with BC and/or OC (first‐degree and/or second‐degree relatives only), histopathologic features of tumors, type of surgery, expression of ER, PR, HER2, Ki67, and prognostic information.

### 2.2 Pathology and immunohistochemistry

All patients who were included in the analysis underwent definitive surgery and had their pathologic specimens reviewed by dedicated breast pathologists. Dedicated breast pathologists performed histopathological examination to determine nuclear grade, estrogen receptor (ER) and progesterone receptor (PR) status, Ki67 labeling index, HER2 status. Assessment of nuclear grade is based on nuclear pleomorphism and mitotic count. A numerical scoring system of 1 to 3 is used to ensure that two factors are assessed independently. ER and PR positivity were determined if ≥10% of nuclei in the tumor stained positive for ER/PR on immunohistochemical analysis. The HER2 staining pattern was divided into 4 groups: 3+ (strong and diffuse staining in >10% of cancer cells), 2+ (moderate and diffuse staining), 1+ (focal staining), and 0. HER2 positive was defined as HER2 staining was 3+ and Fluorescence in situ hybridization (FISH) testing positive of HER2 gene amplification when HER2 staining was 2+. HER2 negative was defined as HER2 staining 0, 1+, and FISH negative when HER2 staining was 2+. Ki67 labeling index was expressed as the percentage of positive cells in each case and a threshold of ≥14% was indicative of a high proliferation index.

### 2.3 Mutation analysis and statistical analysis

We sent genomic DNA samples to FALCO Biosystems Ltd. (Kyoto, Japan), and direct sequencing was performed, by using SBS (Sequencing by Synthesis) Method and MLPA (Multiplex Ligation‐dependent Probe Amplification) Method. All variants detected by direct sequencing were interpreted according to the Myriad Genetics’ criteria.

Patient demographics and clinical characteristics have been tabulated and compared among the two groups, which were defined according to *BRCA* status (non‐carrier and *BRCA1/2* carrier) and between mutation carriers and noncarriers, using chi‐square tests for categorical variables and *t* tests for continuous variables. Univariate and multivariate logistic regression analyses on *BRCA* mutation status (carriers vs. noncarriers) were done to identify factors that were predictive of *BRCA1/BRCA2* mutations in the patients with DCIS. A Cox proportional hazards model was used to estimate the risks of Ipsilateral breast tumor recurrence (IBTR) and contralateral breast tumor recurrence (CBTR). *p* values ≤0.05 were considered statistically significant, and all tests were two‐sided. Statistical analysis was carried out using the SPSS Statistics 23.0 software (IBM^®^).

## 3 RESULTS

### 3.1 *BRCA* mutation with DCIS

Of 325 patients (with or without invasive cancer), 19.1% (62/325) had mutation of *BRCA1* or/and *BRCA*2. There were 49 DCIS in 325 breast cancers, and nine cases out of sixty‐two *BRCA* mutation were DCIS, 2% (*n* = 1) carried a *BRCA1* mutation, and 16.4% (*n* = 8) carried a *BRCA2* mutation (Table 1). The incidence of DCIS among patients with *BRCA* mutation (9/62 cases, 14.5%) was equal to that among patients without *BRCA* mutation (40/263 cases, 15.2%). Incidence of DCIS was 3.0% (1/33) of *BRCA1* mutation carriers and 27.5% (8/29) of *BRCA*2 carriers (*p *= 0.009).

**Table 1:** *BRCA1/2* mutations in DCIS patients: clinical aspects and molecular description

Gene	Nucleotide change	Protein change	Interpretation	Age	BIC name	dbSNP	Ancestry	FH
*BRCA1*	5280C>T	Q1721X	Deleterious	32	—	—	JAP	+
*BRCA2*	8732C>A	S2835X	Deleterious	34	S2835X	rs80359102	JAP	+
*BRCA2*	8251A>G	I2675V	Suspected deleterious	39	—	—	JAP	+
*BRCA2*	8817insA	STOP2868	Deleterious	59	—	—	JAP	+
*BRCA2*	3036del4	STOP959	Deleterious	52	3036del4	rs80359352	JAP	+
*BRCA2*	1506delA	STOP429	Deleterious	34	1506delA	rs80359274	JAP	+
*BRCA2*	4123G>T	E1299X	Deleterious	73	—	—	JAP	+
*BRCA2*	7180C>T	R2318X	Deleterious	47	R2318X	rs80358920	JAP	+
*BRCA2*	1627A>T	K467X	Deleterious	34	K467X	rs80358427	JAP	+

Notes

A: alanine; BRCA1/BRCA2: breast cancer susceptibility genes 1 and 2, respectively; C: cysteine; del: deletion; E: glutamic acid; G: glycine; ins, insertion; K: lysine; Q: glutamine; R: arginine; S: serine; T: threonine; W: tryptophan; X: unspecified amino acid; Y: tyrosine.

Interpretation of Myriad Variant Classification Model: *Deleterious* associated with a significantly increased cancer risk; *Suspected Deleterious* available evidence strongly suggests association with significantly increased cancer risk.

Age in years (range); *BIC* breast cancer information core; Ancestry: *JAP* Japanese; FH family history for breast and/or ovarian cancer: (+): present, (−): absent.

The prevalence of *BRCA1/2* mutations was 17.6% (6/34) in women who had DCIS diagnosed before age 50 years, and patients had *BRCA2* mutations (5/34, 14.7%) more frequently than *BRCA1* mutations (1/34, 2.9%).

Mutations in *BRCA1* was deleterious type (Q1721X). Mutations in *BRCA2*, comprised seven different deleterious type (S2835X, STOP2868, STOP959, STOP429, E1299X, R2318X, K567X) and one suspected deleterious type (I2675V) (Table 1). S2835X, 3036del4, 1506delA, R2318X and K467X was detected once, respectively, and could be searched in the BIC database; I2675V, 8817insA, E1299X was detected in one subject respectively and was far unreported in the BIC database.

Also, another three variants of uncertain significance (VUS) were detected, one in *BRCA1* and two in *BRCA2* gene (Table 2).

**Table 2:** Analysis of missense variants from *BRCA1/2* gene of uncertain significance

Gene	Nucleotide change	Protein change	Interpretation
*BRCA1*	1321G>A	G401E	Uncertain significance
*BRCA2*	9394C>T	H3056Y	Uncertain significance
*BRCA2*	IVS6–2A>G		Uncertain significance

A: alanine; BRCA1/BRCA2: breast cancer susceptibility genes 1 and 2, respectively; C: cysteine; del: deletion; E: glutamic acid; G: glycine; H: histidine; I: isoleucine; V: valine; S: serine; T: threonine; Y: tyrosine.

Interpretation of Myriad Variant Classification Model: *VUS (Variant of Uncertain Significance)* Insufficient evidence to determine if the variant is associated with an increased cancer risk.

#### 3.1.1 Mutation carrier rate by patient demographics and clinical characteristics

The median age of 49 patients at the time of diagnosis was 46 years (ranged from 22 to 73 years). The mean interval between date of diagnosis and date of inclusion in the study was 56.92 months (12–174 months). All patients were born in Japan.

Table 3 lists the characteristics and FH of cancer of 49 DCIS patients included in our study. Overall, patients with a distribution of 18.4% aged ≤35 years and 81.6% >35 years at first diagnosis. Among *BRCA* mutation carriers, 44.4% (4/9) aged ≤35 years and 55.6% (5/9) >35 years. For *BRCA* mutation noncarriers, 12.5% (12/40) aged ≤35 years and 87.5% (35/40) >35 years. Median age of first diagnosis in *BRCA* mutation carriers is 39 years (32–73 years), and 46 years (22–71 years) in noncarriers. About 69.4% (34/49) of patients had an FH of BC, and 69.4% (34/49) had in at least 1 first‐degree or second‐degree relative with BC DX. 10.2% (5/49) had an FH of OC, and 28.6% (14/49) of patients did not report any FH of BC or OC. Patients with an FH of BC and *BRCA* mutation had a higher proportion compared with patients without any FH of BC (26.5% vs. 0%). Patients with an FH of OC and *BRCA* mutation had similar proportions compared with patients who had no FH of OC (20.2% vs. 18.2%). *BRCA* mutation carriers were identified in four of 10 patients (40%) who had ≥2 family members diagnosed with BC compared with five of 24 patients (20.8%) who had a single relative affected by BC.

**Table 3:** Histological and immunohistochemical characterization of *BRCA1/2* noncarriers, *BRCA1/2* carriers and* BRCA2* carriers with DCIS

Characteristic	Non‐*BRCA1/2*		*BRCA1/2*			*BRCA2*		
	*N*(40)	%	*N*(9)	%	*p* value	*N*(8)	%	*p* value
Age at diagnosis (years)								
≤35	5	12.5	4	44.4		3	37.5	
>35	35	87.5	5	55.6	**0.046**	5	62.5	0.116
Ancestry	all Japanese							
FH of BC								
No	15	37.5	0	0		0	0	
Yes	25	62.5	9	100	**0.042**	8	100	**0.044**
FH of 1st or 2nd degree with BC								
No	15	37.5	0	0		0	0	
Yes	25	62.5	9	100	**0.042**	8	100	**0.044**
Total relatives with BC								
0	15	0	0	0		0	0	
1	19	5	5	55.6		5	62.5	
≥2	6	4	4	44.4	**0.013**	3	37.5	**0.027**
1st‐ or 2nd‐degree relatives with BC								
0	25	62.5	2	22.2		2	25.0	
1	14	35	7	77.8		6	75.0	
≥2	1	2.5	0	0	0.057	0	0	0.102
FH of OC								
No	36	90.0	8	88.9		7	87.5	
Yes	4	10.0	1	11.1	1.000	1	12.5	1.000
Nuclear grade								
1	29	74.4	4	44.4		4	50.0	
2	6	15.4	3	33.3		3	37.5	
3	4	10.3	3	22.2	0.218	1	12.5	0.319
2 & 3	10	25.6	5	55.6	0.115	4	50.0	0.215
ER status								
−	10	25.0	1	11.1		0	0	
+	30	75.0	8	88.9	0.662	8	100	**0.042**
PR status								
−	12	30.0	3	33.3		2	25.0	
+	28	70.0	6	66.7	1.000	6	75.0	1.000
HER2 status								
−	28	71.8	4	44.4		3	37.5	
+	11	28.2	5	55.6	0.138	5	62.5	0.101
ER/PR								
−/−	10	25.0	1	11.1		0	0	
+/−	2	5.0	2	22.2		2	25.0	
+/+	28	70.0	6	66.7	0.246	6	75.0	**0.049**
ER/HER2								
−/−	6	15.4	1	11.1		0	0	
−/+	4	10.3	0	0		0	0	
+/−	22	56.4	3	44.3		3	37.5	
+/+	7	17.9	5	55.6	0.115	5	62.5	**0.041**
Ki67 index								
<14%	19	52.8	3	33.3		3	37.5	
≥14%	17	47.2	6	66.7	0.459	5	62.5	0.698
<30%	30	83.3	7	77.8		7	87.5	
≥30	6	16.7	2	22.2	0.651	1	12.5	1.000

Note

*p* values ≤ .05 were considered statistically significant (in bold).

In univariate analyses (Table 3), age at diagnosis was predictive of *BRCA* mutation status (*p *= 0.046), the *BRCA* mutation rate was significantly higher in patients who were younger than 35 years. No significant differences were noted in ER status, PR status, HER2 status or nuclear grade with respect to *BRCA* mutation status. Subgroup analysis showed *BRCA* mutation tumors tended to be ER+/PR+ (6/9, 66.7%) and ER+/HER2+ (5/9, 55.6%), whereas no significant difference compared with *BRCA* mutation noncarriers. Patients who had an FH of BC had a higher risk of having *BRCA* mutations compared with patients who had no FH of BC (26.5% vs. 0%; *p *= 0.042), especially patients who had FH of first‐ or second‐degree BC compared with those who had no FH of first or second degree BC (*p *= 0.042). This same trend was observed among patients who had ≥2 family members with BC compared with those who had fewer relatives with BC (*p *= 0.013), especially patients who had ≥2 first‐ or second degree relatives with BC compared with those who had fewer relatives with BC (*p *= 0.013). *BRCA* mutation status was not associated significantly with an FH of OC (*p *= 1.000). No significant difference was noted in Ki67 index cut off by 14% (*p* = 0.459) or 30% (*p *= 0.651) with respect to *BRCA* mutation status.

Table 4 provides the multivariate logistic regression model for *BRCA* incorporating patient and disease characteristics. Total relatives with BC DX ≥ 2 (odds ratio [OR], 5.128; 95% confidence interval [CI], 1.266–20.763; *p *= 0.022), age at diagnosis ≤35 years (OR 0.149, 95% CI 0.023–0.954, *p *= 0.045) and ER+/HER2+ status (OR 5.034, 95% CI 1.092–23.210, *p *= 0.038) remained as independent, significant predictors for *BRCA* mutation. Specifically, patients who had ≥2 relatives with BC were more likely to have *BRCA* mutations compared with patients who had no relatives with BC. However, the *BRCA* mutation rate did not differ significantly between patients who had ≥2 relatives with BC versus patients who had a single relative with BC (*p *= 0.391).

**Table 4:** Multivariate logistic regression model for breast cancer susceptibility gene mutation status

Variable	OR	95% CI	*P*
ER/HER2	5.034	1.092–23.210	0.038
Age at diagnosis	0.149	0.023–0.954	0.045
Total relatives with BC	5.128	1.266–20.763	0.022

Note

CI: confidence interval.

#### 3.1.2 Histopathology of DCIS in *BRCA2* carriers

Table 3 also listed histological and immunohistochemical characterization of *BRCA2* carriers with DCIS in our study. Univariate analysis showed *BRCA2* mutation has no relationship with age cut off by 35 years, but among aged ≤35 years DCIS patients, *BRCA2* carriers occupied 62.5%, higher than that in aged >35 years patients (37.5%).

##### Histological characteristics

There were no significant differences in nuclear grade between *BRCA2* carriers and noncarriers (*p *= 0.319). We divided patients into two subgroups by Grade 1 and Grade 2&3, there's no significant difference between *BRCA2* carriers and noncarriers (*p *= 0.215). But it's found that *BRCA2* carriers are much higher (50.0%) in Grade 2 and 3 subgroup, which is only 25.6% of *BRCA2* noncarriers. It's probably that DCIS with *BRCA2* is more frequently moderately or poorly differentiated tumors (Grade 2 and 3).

In our study, it's found that a statistically significant difference in ER‐positive tumors among *BRCA2* carriers and noncarriers (*p *= 0.042). About 100% *BRCA2* carriers were ER‐positive, ER‐positive DCIS were more frequent in *BRCA2* carriers. But there's no significant difference in PR‐positive tumors between *BRCA2* carriers and noncarriers (*p *= 1.000). The frequency of PR expression in *BRCA2* mutation DCIS was similar to that in noncarriers. In subgroup analysis, we divided into three subgroups according to ER/PR status (ER−/PR−, ER+/PR− and ER+/PR+). It's found that *BRCA2*‐associated DCIS tended to be more frequently ER+/PR+ status (*p *= 0.049). It's found no differences in the expression of HER2 in *BRCA2* carriers and noncarriers. But Significant difference was also noted in ER/HER2 status subgroup, DCIS with *BRCA2* mutation had higher frequency of ER+/HER2+ status (*p *= 0.041).

No significant difference was noted in Ki67 index cut off by 14% (*p *= 0.698) or 30% (*p *= 1.000) with respect to *BRCA2* mutation status. But when Ki67 index cut off by 30%, 87.5% low proliferation (<30%) was found in *BRCA2* carriers.

The result of multivariate logistic regression model analysis for *BRCA2* mutation showed that ER+/HER2+ status (OR 5.858, 95% CI 1.263–27.167, *p *= 0.024) remained as independent, significant predictors for *BRCA2* mutation. It's suggested that DCIS patients with ER+/HER2+ expression of FH of BC probably have higher risk of *BRCA2* mutation.

#### 3.1.3 Histopathology of DCIS in BRCA1 carriers

In our study, there's only 1 patient in *BRCA1* mutation. This *BRCA1* carrier was Grade 3, triple negative (TN) subtype (ER‐negative, PR‐negative, HER2‐negative) and higher proliferation (ki67 >30%).

### 3.2 Surgery information

Patients underwent definitive surgery either before (*n* = 2) or after (*n* = 47) genetic testing. The surgical intervention was breast‐conserving surgery (BCS) for 51% of patients (*n* = 25), unilateral mastectomy for 42.9% (*n* = 21). 2 carriers (4.1%) underwent prophylactic mastectomy.

### 3.3 Risk factors for Ipsilateral breast tumor recurrence (IBTR) and contralateral breast tumor recurrence (CBTR) in DCIS patients

A total of 25 patients who underwent BCS, 3 (12.0%) suffered IBTR, at a median follow‐up of 64 months (range 16–174 months) for survivors, and recurrent lesions were all IBC. The occurrence of IBTR was higher or tended to be higher in patients with higher NG (NG3) (using Cox univariate analysis, *p *= 0.001). *BRCA* mutation status was not associated significantly with occurrence of IBTR (*p *= 0.865), also no significant difference in patients with FH of BC (*p *= 0.327). Young age (≤35 years), premenopausal status, hormone receptor positive (HR)/HER2 negative and a lack of radiotherapy were not significantly associated with IBTR (*p *= 0.352; *p *= 0.251; *p *= 0.201;* p *= 0.526, respectively) (Table 5). No significant result was found in Cox multivariate analyses.

**Table 5:** Factors influencing IBTR among DCIS patients who underwent lumpectomy, results of cox univariate analysis

Parameter	Total *N*(25)	IBTR *N*(3)	Univariate analysis *p* value
Age at diagnosis (years)			
≤35	4	2	
>35	21	1	0.352
*BRCA* mutation			
−	21	2	
+	4	1	0.865
Menopausal status			
Pre	16	3	
Post	9	0	0.251
FH of BC			
No	8	1	
Yes	17	2	0.327
NG			
1	13	1	
2	9	1	
3	2	1	**0.001**
ER status			
−	5	0	
+	20	3	0.351
PR status			
−	5	0	
+	20	3	0.351
HER2 status			
−	16	2	
+	9	1	0.706
HR/HER2			
+/−	13	2	
Others	12	1	0.201
Adjuvant endocrine therapy			
No	19	2	
Yes	6	1	0.852
Irradiation			
No	11	2	
Yes	14	1	0.526

HER2: human epidermal growth factor receptor 2; HR: hormone receptor; IBTR: ipsilateral breast tumor recurrence; NG: nuclear grade.

*p* values ≤ .05 were considered statistically significant (in bold).

Among all 49 cases, CBTR occurred in nine cases (18.4%), five of which involved invasive ductal carcinomas and four involved DCIS. The median period of CBTR among these nine cases was 36 months (range 12–174 months), after surgery for DCIS. We chose age at diagnosis (cut off by 35 years old), *BRCA* mutation status, menopausal status, nuclear grade, ER status, PR status, HER2 status, FH of BC, ki67 index (cut off by 14% or 30%), subgroup of HR/HER2 status, adjuvant endocrine therapy and irradiation therapy as related risk factors for Cox univariate analysis (Table 6). And we select factors which were *p* value ≤0.5 in univariate analysis as research factors to be used in Cox multivariate proportional hazard general linear models to estimate risk of CBTR. It's found that ER status was an independent risk factor for CBTR on multivariate analysis (hazard ratio 0.085; 95% CI 0.007–1.047; *p* = 0.05) (Table 6), CBTR was higher or tended to be occurred in patients with ER negative.

**Table 6:** Factors influencing CBTR among DCIS patients, results of cox univariate and multivariate analysis

Parameter	Total	CBTR	Univariate analysis	Multivariate analysis		
	*N*(49)	*N*(9)	*p* value	HR	*p* value	95% CI
Age at diagnosis (years)						
≤35	9	0				
>35	39	9	**0.031**			
*BRCA* mutation						
−	39	0				
+	9	9	0.107			
Menopausal status						
Pre	30	7				
Post	18	2	0.476			
FH of BC						
No	15	4				
Yes	33	5	**0.05**			
NG						
1	32	5				
2	9	3				
3	6	1	0.794			
ER status						
−	10	2				
+	38	7	0.349	0.085	**0.05**	0.007–1.047
PR status						
−	14	3	*			
+	34	6	0.092			
HER2 status						
−	31	6				
+	16	2	0.268			
HR/HER2						
+/−	23	4				
Others	24	4	0.833			
Ki67 index (%)						
<14	21	5				
≥14	23	4	0.827			
<30	36	7				
≥30	8	2	0.066			
Adjuvant endocrine therapy						
No	40	8				
Yes	8	1	0.63			
Irradiation						
No	34	7				
Yes	14	2	0.879			

CBTR: contralateral breast tumor recurrence; HER2: human epidermal growth factor receptor 2; HR: hormone receptor; NG: nuclear grade; CI: confidence interval.

*p* values ≤ .05 were considered statistically significant (in bold).

## 4 DISCUSSION

In our study, it indicated an overall 18.4% (17.6% before age 50 years) prevalence of deleterious *BRCA1/BRCA2* mutations in high‐risk women diagnosed with DCIS, supporting the presence of an in situ phase of carcinogenesis in the development of at least some *BRCA*‐associated breast cancer (Arun et al., 2009). One study evaluated the mutation rate in 10,000 consecutive patients who were referred for genetic testing in Myriad Genetic Laboratories (Frank et al., 2002). The prevalence of *BRCA* mutations was 13% in women who had DCIS diagnosed before age 50 years versus 24% in women who had IBC. Hall et al. (2010) conducted a cross‐sectional analysis of the Myriad Genetics *BRCA1/BRCA2* database and reported an overall 5.9% prevalence of *BRCA1/BRCA2* mutations in non‐AJ patients with carcinoma in situ (CIS) (ductal or lobular). But only a few studies published have described the prevalence of *BRCA* mutation status in Japanese women with DCIS. Among mutations detected in *BRCA1*, L63X, and Q934X were reported as founder mutation in Japanese (Ikeda et al., 2001; Lakhani, Easton, & Stratton, 1997; Sekine et al., 2001; Sugano et al., 2008). In our study, Q1721X (c.5280C>T) was detected in only one subject, which has not previously reported nor been listed in the BIC database. Genetic variants of unknown significance G401E (1321G>A) were detected in one subject and also was thus far unreported in the BIC database. In the analysis of *BRCA2*, Ikeda et al. (2001) showed that *BRCA2* 5802delTTAA mutation was considered as common in Japanese breast cancer patients, and Nakamura et al. (Lakhani et al., 1997) reported 5804del4 and R3128X was most frequent mutation. In our study, S2835X, 3036del4, 1506delA, R2318X, I2675V, and 8817insA had been reported in Japanese (Nakamura et al., 2015). K467X (c.1627A>T) was previously reported in a Korean population (Kang et al., 2002), and it's the first time to be detected in a Japanese subject in our study. E1299X (c.4123G>T) was a novel mutational type which was detected in an age 70 years Japanese DCIS patient with luminal B subtype. As for genetic variants of uncertain significance, two mutational types H3056Y (c.9394C>T) and IVS6–2A>G were detected once in our study which were far unreported in the BIC database. IVS6–2A>G had been reported in Japanese (Lakhani et al., 1997), H3056Y (c.9394C>T) was novel mutational type in Japanese. But we did not detect most frequent type of *BRCA*1/2 mutation in our cohort, the reason is perhaps that DCIS is less commonly in breast cancer, a large‐scale cohort study is required to obtain more precise information about founder mutations of DCIS patients in Japan.

In our study, cohort of women with pure DCIS who were referred for genetic risk assessment, we identified the predictive factors for *BRCA1/BRCA2* mutations. Multivariate analysis revealed that ≥2 family members with BC (OR, 5.242) was one of independent predictors for mutation status. Several studies (Frank et al., 1998; Smith et al., 2007; Claus et al., 2005; Hall et al., 2010) have identified an FH of OC and early onset BC as risk factors for *BRCA* mutations among DCIS probands. We found that FH of BC was related to *BRCA* mutation in univariate analyses (*p *= 0.042), but the association failed to reach statistical significance in multivariate analysis, and FH of OC has no relationship with *BRCA* mutation. Hall et al. 2010 determined that women who had early onset DCIS had a significantly increased risk of *BRCA1/BRCA2* mutation compared with women who had late‐onset disease (aged ≥50 years; OR, 1.5; 95% CI 1.1–2.1). This association was higher in women with very early onset disease (age <40 years vs. ≥40 years; OR, 1.8; 95% CI 1.3–2.3). Conversely, Smith et al. (2007) observed similar mutation rates between patients with DCIS who were diagnosed at age <50 years and with those who were diagnosed at a later age. In our study, the *BRCA* mutation rate was significantly higher in patients who were younger than 35 years (*p* = 0.046), but it was also predictive of mutation status in multivariate analysis (OR 0.149, 95% CI 0.023–0.954, *p *= 0.045). ER status, PR status, HER2 status, and Ki67 index (cut off by 14% or 30%) did not differ between *BRCA* mutation carriers and noncarriers. But subgroup analysis showed that ER+/HER2+ status (OR 5.034, 95% CI 1.092–23.210, *p *= 0.038) remained as a significant predictor for *BRCA* mutation. So it's suggested that DCIS patients with total relatives with BC DX ≥2, age at diagnosis ≤35 years and ER+/HER+ status have high risk of *BRCA* mutation and could be recommended to receive genetic counseling and *BRCA* testing.

*BRCA1* tumors are more frequently poorly differentiated (grade 3) carcinomas. The proportion of grade 3 carcinomas ranged from 66% to 100% in different series, (Eerola et al., 2005; Lakhani et al., 1997; Lynch, Holden, Buys, Neuhausen, & Gaffney, 1998) while the proportion of grade 3 tumors in age‐matched sporadic tumors ranged from 15% to 55% (Agnarsson et al., 1998; Eerola et al., 2005; Lakhani et al., 1997, 2000 ; Lynch et al., 1998). In our study, there's only 1 *BRCA1* mutation, it has Grade 3, TN subtype and higher proliferation (ki67 > 14%). It perhaps indicated that DCIS occurring in carriers of *BRCA1* mutations are also more likely to be ER‐negative, PR‐negative, HER2 receptor‐negative, and have a basal phenotype.

*BRCA2* tumors are more frequently moderately or poorly differentiated carcinomas (grades 2 and 3) (Agnarsson et al., 1998; Lakhani et al., 1997, 2000 ; Lynch et al., 1998). In our study, no significant differences were found in grade between *BRCA2* carriers and noncarriers (*p *= 0.319). But it's found that *BRCA2* carriers are much higher in Grade 2 and 3 compared to that of *BRCA2* noncarriers. It's similar to *BRCA2* carriers in invasive carcinomas. In *BRCA2* tumors, in contrast to *BRCA1* tumors, it has been shown that the percentage of ER‐positive cases. Foulkes et al. (2004) analyzing tumors from *BRCA2* positive patients <45 years, found a statistically significant difference in the percentage of ER‐positive tumors among *BRCA2* carriers and noncarriers. The percentage of ER‐positive tumors was higher in *BRCA2* tumors; however, there was no difference found in a subanalysis of tumors from patients >55 years. Our data indicated that *BRCA2* carriers with DCIS were tend to be ER‐positive (*p *= 0.042) no matter with the age of patients, and subgroup analysis showed BRCA2 tumors with DICS was higher in expression of ER+/PR+ (*p *= 0.049). Data on HER2 expression in *BRCA2*‐associated tumors vary from series to series, probably as a consequence of differences in the techniques employed. We found no significant difference between HER2 status and *BRCA2* mutation, but subgroup analysis suggested that ER+/HER2+ could be an independent risk factor for *BRCA2* mutation, and it's also one of independent predictive factors for *BRCA* mutation in our study. Since *BRCA2* has a major role in DNA repair, its suppression is thought to induce unrepaired DNA lesions, which cause cell cycle arrest by activating checkpoint signaling. So we also found *BRCA2* carriers with DCIS were tend to be lower proliferation (<30%), whereas has no significant difference.

The proportion of DCIS among surgically resected breast cancers is reported to be 20% in Western countries and nearly 10% in Japan (Burstein, Poluak, Wong, Lester, & Kaelin, 2004; Cancer Statistics in Japan, 2013; Ernster et al., 2003). Pure DCIS in itself is not a life‐threatening disease, and the local recurrences if that appear as DCIS do not influence the overall survival rate of patients. True DCIS will theoretically not metastasize to regional lymph nodes or relapse in a distant organ, and thus the management of DCIS patients focuses on local control of the primary lesion and early detection and treatment of both IBTR and CBTR (Fisher et al., 2002, 1999 ). *BRCA1/BRCA2* mutations have been shown to indicate a higher susceptibility to develop BC. Individuals who carry one of these mutations have a 43%–84% risk of developing BC, and up to a 65% risk for CBC (Finkelstein et al., 2006; Ford et al., 1998). But in our study, *BRCA* mutation status was not associated significantly with occurrence of IBTR. It indicated that, BCS might be a treatment option for DCIS patients with *BRCA* mutation, for they may be more sensitive to radiation (Garcia‐Etienne et al., 2009; Kirova et al., 2010). But we need additional data of more research samples to support this conclusion. It's also found that CBTR rate was higher or tended to be higher in DCIS patients with ER negative, but no relationship with *BRCA* mutation status. Further study was needed to discuss whether *BRCA* mutation is the independent factor for CBTR in DCIS patients, and it's very important for DCIS women with *BRCA* mutation whether or not to choose contralateral prophylactic mastectomy.

## 5 CONCLUSION

DCIS is equally as prevalent in patients who were *BRCA* mutation carriers as in high familial‐risk women who were noncarriers, but occurs at earlier age. *BRCA2* carriers have higher incidence in DCIS than that of *BRCA1* carriers, and tend to be higher grade and more frequently ER positive and lower proliferation. Total relatives with BC DX ≥2, age at diagnosis ≤35 years and ER+/HER2+ might be independent predictors for *BRCA* mutation in Japanese women with DCIS and patients of these risk factors should be recommended to receive genetic counseling and *BRCA* testing.

## CONFLICT OF INTEREST

None declared.
